# Recurring staphylococcal scalded skin syndrome in a very low birth weight infant: a case report

**DOI:** 10.4076/1752-1947-3-7313

**Published:** 2009-08-12

**Authors:** Carola EPM Duijsters, Feico JJ Halbertsma, René F Kornelisse, Niek LA Arents, Peter Andriessen

**Affiliations:** 1 Department of Pediatrics, Division of Neonatology, Máxima Medical Center Veldhoven, The Netherlands; 2 Department of Pediatrics, Division of Neonatology, Erasmus MC-Sophia Children's Hospital, Rotterdam, The Netherlands; 3 Laboratories of Pathologic Anatomy and Medical Microbiology Veldhoven, The Netherlands

## Abstract

**Introduction:**

Staphylococcal scalded skin syndrome is an extensive desquamative erythematous condition caused by exfoliative toxins of *Staphylococcus aureus*. This disease usually affects neonates and generally responds rapidly to antibiotic therapy.

**Case presentation:**

We describe the case of a premature baby boy, weighing 1030 g, born after 26 6/7 weeks gestation, who developed two episodes of Staphylococcal scalded skin syndrome on days 19 and 48 of life. Cultures obtained during the first period did not reveal *Staphylococcus aureus*, but diagnosis was based on typical clinical grounds.

Although the initial diagnosis was irritation by the fixation material of a nasal continuous positive airway pressure tube, the infant showed rapidly progressing skin blistering and exfoliation, characteristic of Staphylococcal scalded skin syndrome. After administration of antibiotic treatment, complete recovery was seen. In the second period, diagnosis of Staphylococcal scalded skin syndrome was made clinically and confirmed by results of microbiologic investigations. *Staphylococcus aureus* was cultured from the nose, skin lesions and the pharynx. The strain appeared to produce exfoliative toxin A. The clinical response to similar antibiotic treatment was identical to the first period of Staphylococcal scalded skin syndrome.

**Conclusion:**

This case report discusses an unusual presentation of recurring Staphylococcal scalded skin syndrome in a baby with a very low birth weight.

## Introduction

Staphylococcal scalded skin syndrome (SSSS) is a rapidly expanding exfoliative disease of the skin characterized by blistering and epidermal peeling. The disease is induced by exfoliative toxins (ET) of *Staphylococcus aureus* and typically occurs in newborn babies with an onset between the first 3 and 16 days of life [1]. In this case report we discuss an unusual presentation of recurring SSSS in a baby with a very low birth weight (VLBW). Furthermore, we emphasize the importance of early diagnosis of SSSS because of the risk of nosocomial spread to other patients in the Neonatal Intensive Care Unit (NICU), the morbidity and the increased risk of mortality in children [2].

## Case presentation

A baby boy weighing 1030 g was born at 26 6/7 weeks gestation by vaginal delivery. Pregnancy was uncomplicated until 2 days before delivery when spontaneous rupture of membranes occurred. The mother received tocolytic treatment but labour continued and the baby was delivered. Apgar scores were 2, 6 and 9 at 1, 5 and 10 minutes after birth, respectively.

The infant was transported to the NICU with nasal continuous positive airway pressure (CPAP). Because rupture of membranes existed for more than 48 hours, antibiotics (amoxicillin-clavulanic acid and gentamicin) were started. Blood culture remained negative, but group B streptococcus was cultured from the infant's ear and the placenta. We found no clues of infection clinically or in laboratory tests. Amoxicillin-clavulanic acid was continued for 7 days and gentamicin for 3 days.

**Figure 1:**
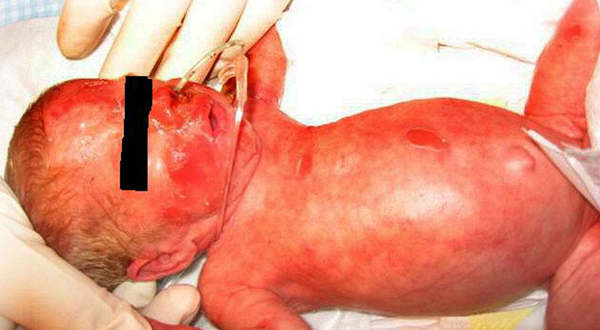
**Red skin lesions with epidermal peeling on the face and body of a baby boy with a very low birth weight**.

On day 19 of life, a 5 mm red skin lesion with epidermal peeling was noticed on the left nostril were the nasal CPAP tube was fixed. Within 6 hours the lesions expanded and new lesions of blisters and exfoliation appeared on shoulders, trunk and the peri-umbilical region. This left an erythematous and moist surface (Fig. 1). The infant was sensitive to manipulation and extremely irritable. The clinical appearance was interpreted as SSSS and blood culture, culture of a blister, and swabs of the nose and perineum were obtained. We started a combination therapy of intravenous flucloxacillin (75 mg/kg in 3 doses) for 10 days, local administration of fusidic acid and adequate pain treatment with intravenous acetaminophen and morphine following premature infant pain profile scores. The patient was isolated. One day after initiation of antibiotic therapy, the lesions ceased to increase or expand. Complete recovery of the affected skin was seen within 11 days. Blood culture remained negative, as were swabs of the nose and perineum. Culture of the skin lesion revealed coagulase-negative staphylococci. Skin biopsy was not performed. The patient was discharged to the pediatric ward approximately one month after birth.

On day 48 of life, suspicion of SSSS arose again and the patient was transferred back to the NICU. The infant developed discrete exfoliation of the nose and the distal part of digits I and III. Within 24 hours, the scrotum, all fingers and the inguinal and peri-umbilical regions were affected. Again we noticed rapidly expanding skin lesions with blistering, epidermal peeling and erythroderma. The boy had no clinical signs of infection, but became agitated when touched. After obtaining cultures from the skin (digits I and III), the nose, the pharynx and the blood, intravenous flucloxacillin was administered for 15 days. Supportive treatment consisted of adequate analgesic treatment, aseptic care and isolation of the patient. As in the first episode of SSSS, no further increase in the lesions was noticed after 36 hours and complete resolution appeared after 1 week. *S. aureus* was cultured from the nose, the fingers and the pharynx. Blood culture was again sterile. The material was sent to The National Institute for Public Health and the Environment for polymerase chain reaction analysis of the exfoliative toxin genes of the strain. The *S. aureus* strain was shown to possess the *eta* gene for Exfoliative Toxin A (ET-A). This confirmed our clinical diagnosis of SSSS. The course of further treatment of the patient was uneventful and discharge to home took place on day 74. Follow-up in our out-patient clinic till 6 months after discharge was completely satisfactory without any relapse of symptoms of SSSS.

## Discussion

SSSS is a clinical manifestation of infection caused by exfoliative toxin producing Staphylococci, usually phage II *S. aureus* strains. These toxins, especially ET-A and ET-B, spread hematogenously and cause erythema, blistering and superficial scalding of the skin by targeting the protein desmoglein I in the zona granulosa of the epidermis [3]. Of the *S. aureus* strains 5% to 6% produce ET, with over 80% of the ET as ET-A. In this case report, investigations for exfoliative toxins were positive for ET-A. This result confirmed our clinical diagnosis of SSSS.

The differential diagnosis of skin exfoliation in neonates includes SSSS, bullous impetigo, chemical burns, drug or viral-induced toxic epidermal necrolysis, epidermolysis bullosa, bullous mastocytosis and neonatal pemphigus [4]-[6].

Diagnosis of SSSS is mainly based on clinical criteria and only confirmed by exotoxin producing *S. aureus*. Diagnosis based on blood or tissue cultures often occurs later than clinical diagnosis. Therefore, fast recognition of this disease is required to start systemic antibiotic treatment with β-lactamase-resistant penicillin at an early stage. Although this treatment is easy and effective within 24 to 36 hours, mortality is still 3% to 11% in children [2] and over 50% in adults [3]. Besides antibiotic therapy and supportive and aseptic skin care, adequate analgesic treatment (morphine), minimal handling and appropriate management of fluid and electrolyte balance are necessary [5,7]. To prevent outbreaks of SSSS involving a large number of newborn babies in neonatal wards, it is important to isolate the infected infant in an incubator [8].

Epidemiological studies showed a prevalence of 3% to 6% of toxin producing *S. aureus* carriage, with a prevalence of 3% in antenatal women [8]. Hargiss et al. [9] reported that up to 60% of neonates discharged from hospital may be nasal carriers of *S. aureus*. Although these data may suggest a high incidence of SSSS, Mockenhaupt et al. [2] calculated an overall incidence between 0.09 and 0.13 cases per million habitants per 5 year with 95% confidence intervals of 0 to 4. SSSS predominantly occurs in neonates, with onset between 3 and 16 days and is less common in preterm infants [1]. Factors that may be responsible for the higher incidence in neonates in comparison to adults include renal immaturity, resulting in diminished clearance of toxins and a lack of specific antibodies against staphylococcal toxins [7].

Less than 10 cases of SSSS in infants with VLBW have been reported in the literature [4]-[6,10]-[12]. Recurrence of SSSS appears to be even more uncommon, especially among preterm neonates. Dobson et al [13] describe an adult patient who developed SSSS 8 days following the cessation of antibiotics for a chest infection and pressure sores. Diagnosis was made on a clinical basis and the patient was treated with intravenous cefuroxime. Recrudescence of the disease appeared after switching to oral cefaclor [13]. Rieger-Fackeldey et al. [14] report SSSS in an extremely low birth weight infant with a relapse 4 weeks after the primary infection. Another case report mentions SSSS in two male siblings aged 5 and 10 years old. This condition recurred in the same children within a period of about 12 months [15]. Our case report in which we describe a preterm infant with a birth weight of 1030 g with two episodes of SSSS on days 19 and 48 of life seems to be unique in the literature.

## Conclusion

We describe an unusual case of recurring SSSS in a VLBW infant and underline the necessity of early diagnosis and treatment of this disease.

## Consent

Written informed consent was obtained from the patient's parents for publication of this case report and any accompanying images. A copy of the written consent is available for review by the Editor-in-Chief of this journal.

## Competing interests

The authors declare that they have no competing interests.

## Authors' contributions

All authors contributed to acquisition of case details and their analysis and interpretation. CD wrote the first draft of the manuscript. FH, RK, NA and PA revised the manuscript. All authors have given final approval of this manuscript to be published.

## References

[B1] Dancer SJ Simmons NA Poston SM Noble WC Outbreak of staphylococcal scalded skin syndrome among neonates J Infect 1988 16 87 103 10.1016/S0163-4453(88)96249-4 3367061

[B2] Mockenhaupt M Idzko M Grosber M Schopf E Norgauer J Epidemiology of staphylococcal scalded skin syndrome in Germany J Invest Dermatol 2005 124 700 703 10.1111/j.0022-202X.2005.23642.x 15816826

[B3] Cribier B Piemont Y Grosshans E Staphylococcal scalded skin syndrome in adults. A clinical review illustrated with a new case J Am Acad Dermatol 1994 30 319 324 10.1016/S0190-9622(94)70032-X 8294590

[B4] Coleman JC Dobson NR Diagnostic dilemma: extremely low birth weight baby with staphylococcal scalded-skin syndrome or toxic epidermal necrolysis J Perinatol 2006 26 714 716 10.1038/sj.jp.7211599 17066068

[B5] Hutten M Heimann K Baron JM Wenzl TG Merk HF Ott H Staphylococcal scalded skin syndrome as a harbinger of late-onset staphylococcal septicaemia in a premature infant of very low birth weight Acta Derm Venereol 2008 88 416 417 18709325 10.2340/00015555-0447

[B6] Makhoul IR Kassis I Hashman N Sujov P Staphylococcal scalded-skin syndrome in a very low birth weight premature infant Pediatrics 2001 108 E16 10.1542/peds.108.1.e16 11433095

[B7] Haveman LM Fleer A de Vries LS Gerards LJ Congenital staphylococcal scalded skin syndrome in a premature infant Acta Paediatr 2004 93 1661 1662 10.1080/08035250410022369 15918230

[B8] Ladhani S Evans RW Staphylococcal scalded skin syndrome Arch Dis Child 1998 78 85 88 10.1136/adc.78.1.85 9534685 PMC1717447

[B9] Hargiss C Larson E The epidemiology of Staphylococcus aureus in a newborn nursery from 1970 through 1976 Pediatrics 1978 61 348 353 643410

[B10] Haveman LM Fleer A Gerards LJ Staphylococcal scalded skin syndrome in two very low birth weight infants J Perinat Med 2003 31 515 519 10.1515/JPM.2003.078 14711108

[B11] Saiman L Jakob K Holmes KW Whittier S Garzon MC Rago JV Molecular epidemiology of staphylococcal scalded skin syndrome in premature infants Pediatr Infect Dis J 1998 17 329 334 10.1097/00006454-199804000-00012 9576389

[B12] Kapoor V Travadi J Braye S Staphylococcal scalded skin syndrome in an extremely premature neonate: a case report with a brief review of literature J Paediatr Child Health 2008 44 374 376 10.1111/j.1440-1754.2008.01316.x 18476932

[B13] Dobson CM King CM Adult staphylococcal scalded skin syndrome: histological pitfalls and new diagnostic perspectives Br J Dermatol 2003 148 1068 1069 10.1046/j.1365-2133.2003.05323.x 12786851

[B14] Rieger-Fackeldey E Plano LR Kramer A Schulze A Staphylococcal scalded skin syndrome related to an exfoliative toxin A- and B-producing strain in preterm infants Eur J Pediatr 2002 161 649 652 10.1007/s00431-002-1080-z 12447663

[B15] Machang'u RS Mgode G Gisakanyi N Recurrent staphylococcal scalded skin syndrome in children: report of two cases East Afr Med J 1997 74 603 604 9487441

